# Evaluation of the Prognostic Value of Long Noncoding RNAs in Lung Squamous Cell Carcinoma

**DOI:** 10.1155/2022/9273628

**Published:** 2022-01-13

**Authors:** Xiaoting Zhang, Yue Su, Xian Fu, Jing Xiao, Guicheng Qin, Mengli Yu, Xiaofeng Li, Guihong Chen

**Affiliations:** ^1^Shenzhen Bao'an District Songgang People's Hospital, Shenzhen, China; ^2^School of Pharmaceutical Sciences, Guangzhou Medical University, Guangzhou, China; ^3^Department of Laboratory Medicine, Peking University Shenzhen Hospital, Shenzhen, China

## Abstract

Lung squamous cell carcinoma (LUSC) is the most common type of lung cancer accounting for 40% to 51%. Long noncoding RNAs (lncRNAs) have been reported to play a significant role in the invasion, migration, and proliferation of lung cancer tissue cells. However, systematic identification of lncRNA signatures and evaluation of the prognostic value for LUSC are still an urgent problem. In this work, LUSC RNA-seq data were collected from TCGA database, and the limma R package was used to screen differentially expressed lncRNAs (DElncRNAs). In total, 216 DElncRNAs were identified between the LUSC and normal samples. lncRNAs associated with prognosis were calculated using univariate Cox regression analysis. The overall survival (OS) prognostic model containing 10 lncRNAs and the disease-free survival (DFS) prognostic model consisting of 11 lncRNAs were constructed using a machine learning-based algorithm, systematic LASSO-Cox regression analysis. We found that the survival rate of samples in the high-risk group was lower than that in the low-risk group. Results of ROC curves showed that both the OS and DFS risk score had better prognostic effects than the clinical characteristics, including age, stage, gender, and TNM. Two lncRNAs (LINC00519 and FAM83A-AS1) that were commonly identified as prognostic factors in both models could be further investigated for their clinical significance and therapeutic value. In conclusion, we constructed lncRNA prognostic models with considerable prognostic effect for both OS and DFS of LUSC.

## 1. Introduction

Lung cancer is one of the most common types of cancer. In 2018, lung cancer accounted for 11.6% of global cancer [1], and more than 1,600,000 new cases are diagnosed yearly [2]. Due to its indistinct early symptoms, it is often diagnosed in the middle or late stages, which usually leads to a very poor prognosis [3]. Non-small-cell lung cancer (NSCLC) accounts for more than 80% of total lung cancer, including lung adenocarcinoma (LUAD), lung squamous cell carcinoma (LUSC), and large cell carcinoma (LCLC), among which LUAD and LUSC are the most prevalent ones [4, 5]. Despite advances in the treatment methods of LUSC, the mortality is still high, and the 5-year overall survival (OS) rate of LUSC patients with clinical I and II stages is about 40%. Notably, the 5-year OS rate for the stage III–IV LUSC patients is less than 5% [6, 7]. However, the basic methods for assessing the diagnosis and prognosis of LUSC are based on disease stage and histological grade.

Long noncoding RNAs (lncRNAs) are a type of noncoding RNAs of over 200 base pairs with limited protein-coding potential [8]. Recently, an increasing number of lncRNAs have been identified in humans, and the number continues to rise [9–11]. However, only a small fraction of human lncRNAs have been comprehensively investigated and functionally annotated, resulting in the majority of the rest being still annotated as unknown functions [12]. In recent years, numerous studies have indicated that the dysregulation of certain lncRNAs plays an important role in a variety of tumors [13–16]. Researchers have paid increasing attention to the potential of lncRNAs in cancer diagnosis and prognosis because the aberrant expression of lncRNAs is associated with the cancer onset and progression [17–19]. Therefore, finding effective prognostic lncRNA biomarkers to prompt therapy and improve the patient survival rate has great significance in cancers.

Recent developments of sequencing and omics technologies provide the opportunity to perform large-scale measurements of diseases at the expression level. High-dimension data problems including prognostic analysis can be addressed using machine learning algorithms. In this study, LUSC data with a large sample size were downloaded from TCGA database [20] and were systematically integrated and analyzed based on bioinformatics methods including differentially expressed gene analysis (DEGA), Gene Ontology (GO) enrichment analysis, and Kyoto Encyclopedia of Genes and Genomes (KEGG) pathway analyses. Then, we constructed OS and DFS prognostic models of LUSC using least absolute shrinkage and selection operator (LASSO) and Cox regression analysis and explored the key lncRNAs as potentially valuable prognosticators associated with LUSC.

## 2. Materials and Methods

### 2.1. Data Collection

**Figure 1:**
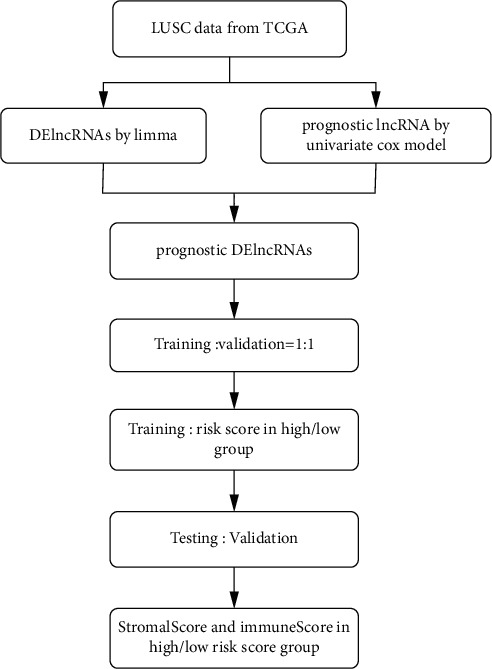
The workflow of this study.

The RNA-seq data of 543 samples and their corresponding clinical information, including 494 LUSC patients and 49 normal controls, were collected from TCGA database via UCSC Xena (https://xenabrowser.net/hub/). lncRNA-RNA interaction relationships were obtained from starBase 3.0 (http://starbase.sysu.edu.cn/index.php), and these RNAs were used as potential target genes for lncRNAs. The clinical characteristics of LUSC patients are listed in Table 1, and the research procedure is indicated in Figure 1.

### 2.2. Differential Analysis of lncRNA

The limma R package was used to identify the differentially expressed lncRNAs (DElncRNAs) between the LUSC and normal samples. Absolute value of fold change (FC) > 2 and FDR-adjusted *P* value <0.05 were used as thresholds. The ggplot2 package was used to draw the volcano plot of lncRNA expression, and the pheatmap package was used to plot the heatmap of the identified DElncRNAs.

### 2.3. Screening of the Prognostic lncRNA

Univariate Cox regression analysis was applied for the clinical data and the lncRNA expression data using the survival R package. lncRNAs related to OS and DFS were separately screened with the *P* value of 0.05 as the threshold. Then, a machine learning-based algorithm, LASSO-Cox regression analysis, was used to screen a panel of lncRNAs that were significantly related to OS and DFS. Next, the LUSC samples were randomly divided into a training set and a test set at a ratio of 1 : 1, and 10-fold cross-validation was performed to tune lncRNAs related to OS and DFS in the training set.

### 2.4. Functional Enrichment Analysis

Target genes of lncRNAs were obtained from the starBase 3.0 database [21, 22]. These target genes were further analyzed using Gene Ontology (GO) [23] and Kyoto Encyclopedia of Genes and Genomes (KEGG) pathway [24] functional enrichment analyses via the clusterProfiler R package [25]. An FDR-adjusted *P* value <0.05 was considered to be statistically significant. GO enrichment analysis was performed for ontologies of biological process (BP), cellular component (CC), and molecular function (MF). In order to compare the difference of immune and stromal scores in the high- and low-risk groups, the expression signature of LUSC samples was calculated by the estimate package in R.

### 2.5. Construction of the lncRNA Prognostic Model and Survival Analysis

The univariate Cox regression method was separately conducted to select lncRNAs related to OS and DFS using the survival package in R. Furthermore, a multivariate Cox regression analysis was performed to confirm their independence, and log-rank *P* value <0.05 was considered as statistically significant. Subsequently, the prognostic risk score model of OS and DFS was established, respectively, with the use of survival-related lncRNAs (formula (1)) by using the survival R package. The prognostic risk score model of OS and DFS was as follows: Risk_score = *∑ coef*_*i*_ *∗* *lncRNA*_*i*_, where *coef*_*i*_ is the coefficient of the *i* lncRNA in multivariate Cox regression analysis and *lncRNA*_*i*_ is the expression level.

LUSC samples were further divided into the high-risk group and low-risk group according to their median risk score [26, 27]. The survival R package was then separately used to map survival curves of the high-risk group and low-risk group. In addition, a log-rank test was used to estimate the significance between survival curves and further analyze the difference of survival between the two groups.

### 2.6. Assessment of the Prognostic Predictive Risk Models

To validate the prediction accuracy of the prognostic risk model, the receiver operating characteristic (ROC) curves were used to compare the high-risk and low-risk LUSC patients. Furthermore, to verify whether the lncRNA prognostic model was an independent prognostic factor, the univariate and multivariate Cox regression tests were conducted for OS and DFS separately, using the risk score and clinical features (such as stage, gender, age, and TNM) in the first, third, and fifth year by calculating the area under the ROC curves (AUCs).

## 3. Results

### 3.1. Screening of Differentially Expressed lncRNAs

**Figure 2:**
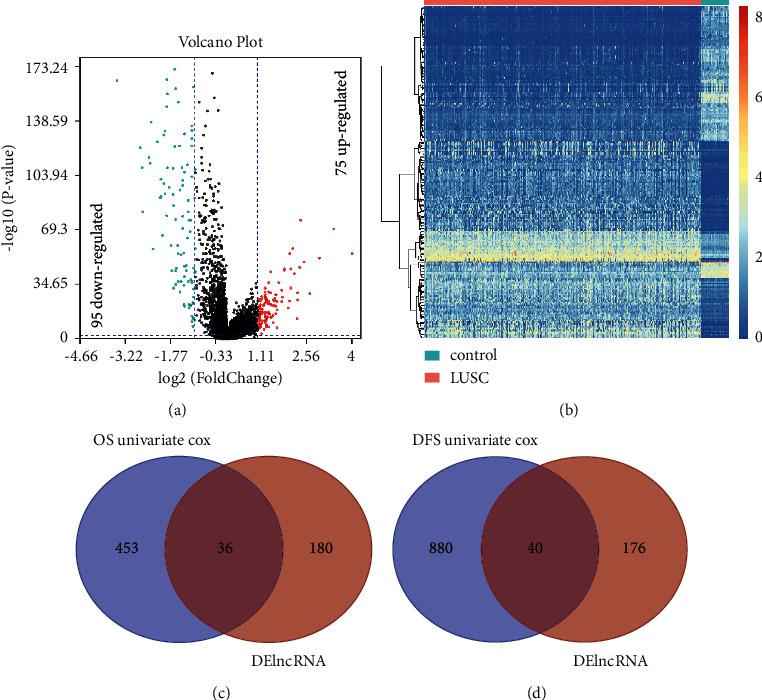
(a) Volcano plot of the screened DElncRNA. (b) Heatmap showing the expression abundance of the DElncRNAs. (c) Venn diagram of the DElncRNAs and OS-related lncRNAs in LUSC. (d) Venn diagram of the DElncRNAs and DFS-related lncRNAs in LUSC.

To obtain differentially expressed lncRNAs (DElncRNAs) between LUSC and normal samples, the expression signature of lncRNAs in 494 LUSC patients and 49 normal samples was obtained from TCGA database and was screened by the limma R package. A two-fold change and an FDR-adjusted *P* value of 0.05 were set as the thresholds for DElncRNA identification. A total of 216 DElncRNAs were screened, including 95 downregulated and 75 upregulated DElncRNAs (Figure 2(a)). The expression abundance of these DElncRNAs was illustrated in a heatmap (Figure 2(b)).

### 3.2. Screening of lncRNAs Related to Prognosis

In order to get lncRNAs related to prognosis, a univariate Cox regression analysis was applied to compare the clinical features (including the OS, DFS, and corresponding survival status) of the LUSC samples and normal samples from TCGA database. Using a *P* value of 0.05 as the threshold, we obtained 489 lncRNAs significantly related to OS and 920 lncRNAs positively related to DFS, among which there were 36 DElncRNAs related to OS (Figure 2(c)) and 40 DElncRNAs related to DFS, respectively (Figure 2(d)). In other words, these overlapped lncRNAs were both differentially expressed and survival related.

**Figure 3:**
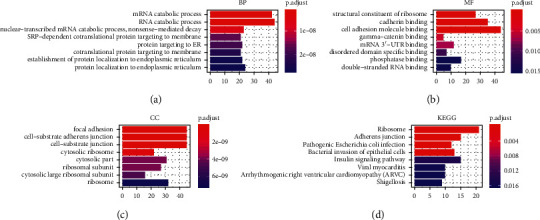
Functional enrichment analysis of GO and KEGG. Functional categories including biological process (BP), molecular function (MF), and cellular component (CC) were analyzed, respectively. GO, Gene Ontology; KEGG, Kyoto Encyclopedia of Genes and Genomes.

The starBase 3.0 database was used to identify the target genes of the OS- and DFS-related lncRNAs. Functional enrichment analysis showed that the target genes were enriched in several biological processes, including protein localization to the endoplasmic reticulum, SRP-dependent cotranslational protein targeting the membrane, cotranslational protein targeting the membrane, and mRNA catabolic process (Figure 3(a)). Structural constituent of the ribosome, cell adhesion molecule binding, and cadherin binding are the main molecular functions these lncRNAs are involved in (Figure 3(b)). The products of these targeting genes were located in the cell-substrate junction and ribosome-related compartment (Figure 3(c)). Also, the results of KEGG enrichment analysis showed that the target genes are mainly involved in pathways of the ribosome, pathogenic *Escherichia coli* infection, and the insulin signaling pathway (Figure 3(d)).

Many previous works have studied the interactions between ribosomes and lncRNAs using ribosome profiling techniques, with a primary focus on probing lncRNAs interacting with ribosomes related to protein synthesis as well as other unclear biological functions. Several cytoplasmic lncRNAs have recently been reported to interact with ribosomes. In footprinting experiments to map ribosome-bound transcripts genome-wide, a considerable number of lncRNAs were identified directly involved in the translation machinery.

### 3.3. Construction of Prognostic Models

To build a prognostic model, samples with OS information were randomly divided into the training set and the test set at a ratio of 1 : 1. In the training set, LASSO-Cox regression analysis was used to calculate the 36 DElncRNAs related to OS, and 10 of them were considered as independent markers with significant prognostic value for LUSC (Figure S1). The coefficients and DElncRNAs are described as follows:

(1) $$\begin{array}{c} OS\_risk_{score}=AC013457.1*0.156001+AC124067.2*\left(-0.068019\right)+AP001189.1\\ {}*0.697599+AP002360.1*\left(-0.168550\right)+BANCR*\left(-0.569796\right)\\ +LINC00519*\left(-0.042434\right)+LINC01807*\left(0.288462\right)\\ +MIR3945HG*0.278341+\left(FAM83A-AS1\right)*\left(-0.007179\right)\\ +\left(POU6F2-AS2\right)*\left(-0.106658\right). \end{array}$$

Similarly, for samples with DFS information, they were randomly split into a training set and a test set of fifty-fifty. 40 DElncRNAs related to DFS were filtered in the training set, and 11 DElncRNAs significantly related to DFS were obtained (Figure S2). The coefficients and DElncRNAs of the DFS prognostic model are described as follows:

(2) $$\begin{array}{c} DF\;S\_risk_{score}=\left(FAM83A-AS1\right)*0.23834+AC010275.1*\left(-0.08770\right)\\ +AC015922.3*0.27021+AL132712.1*\left(-0.15284\right)+LINC00261\\ {}*0.20817+LINC00511*\left(-0.02819\right)+LINC00519*\left(-0.11217\right)\\ +LINC01980*\left(-0.07532\right)+TEM99*\left(-0.49311\right)+MYOSLI\;D\\ {}*0.34051+\left(NUP50-DT\right)*\left(-0.34856\right). \end{array}$$

### 3.4. Survival Analysis of the Prognostic Model

**Figure 4:**
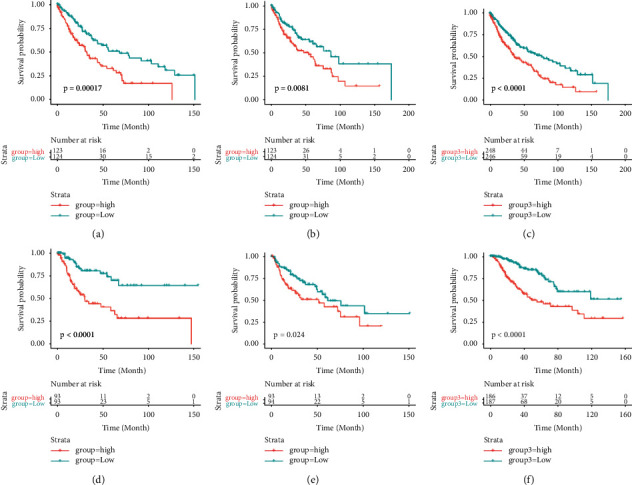
OS survival curve of high- and low-risk groups in the training set (a), test set (b), and entire set (c). DFS survival curve of high- and low-risk groups in the training set (d), test set (e), and entire set (f).

To conduct the survival characteristics of the prognostic model related to OS, the risk scores were calculated for each sample according to the abovementioned formulas. Samples were categorized into high-risk and low-risk groups by the median of the prognostic risk scores, and the survival curve was mapped. The survival differences were calculated using the log-rank test. It revealed that, in the training set, the survival rate of samples in the high-risk group was significantly lower than that in the low-risk group (*P* value <0.05, log-rank test) (Figures 4(a)–4(c)). Similar results were also observed in the test set and the entire set. We can draw the same conclusion for the DFS prognostic model when the same analysis procedure was performed. The survival rate of samples in the high-risk group was significantly shorter than that in the low-risk group in the training set, test set, and entire dataset (*P* value <0.05, log-rank test) (Figures 4(d) and 4(e)).

### 3.5. Correlation Analysis of the Prognostic Model and Clinical Characteristics

**Figure 5:**
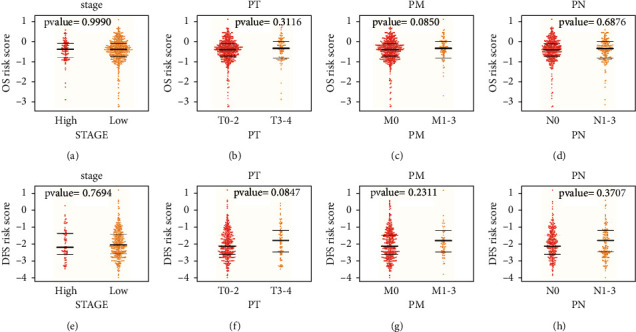
The distributions of the risk scores for OS in stage (a), PN (b), PM (c), and PT (d). The distributions of the risk score for DFS in stage (e), PN (f), PM (g), and PT (h). The bold line in the middle indicates the median, while the other two lines represent the first and third quantile of the distribution, respectively.

We then evaluated the correlations between the risk scores and the clinical characteristics, including age, stage, gender, and TNM. The differences of risk score between groups of age, stage, gender, and TNM were calculated by the log-rank test. We observed that neither the OS risk score nor the DFS risk score has a significant correlation with the clinical characteristics (Tables S1 and S2), and no significant difference was found between groups of the stage (high and low), T (T0–2 and T3–4), N (N0 and N1–3), and M (M0 and M1–3) (Figure 5), where T refers to the size and extent of the primary tumor, N refers to the number of nearby lymph nodes that have cancer, and M refers to whether the cancer has metastasized. These results revealed that OS risk score and DFS risk score were independent predictors of survival risk for the clinical factors.

### 3.6. Evaluation of the Efficiency of Prognostic Models

**Figure 6:**
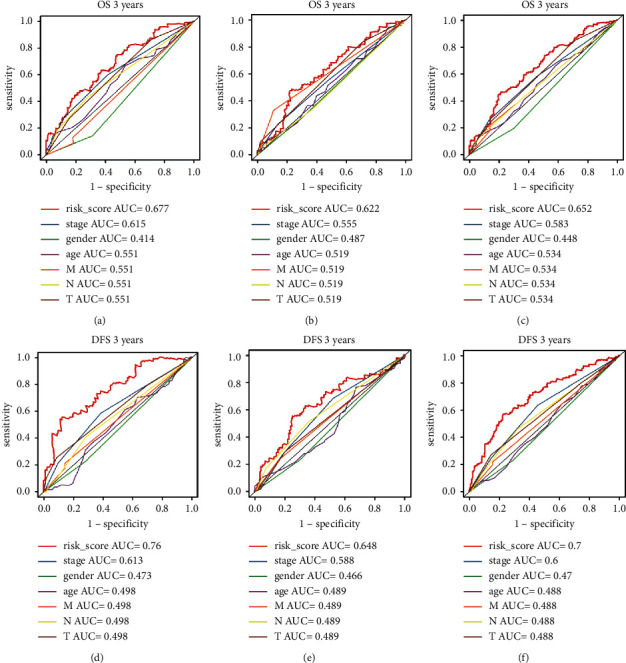
Performance evaluation of the models. ROC curves of the OS prognostic model in the training set (a), test set (b), and entire set (c) in the third year. ROC curves of the DFS prognostic model in the three sets (d–f).

The prognostic model of OS was used to compare with stage, gender, age, and TNM in the third year. The AUCs of the OS prognostic model in the training set, test set, and entire set were consistently higher than those of the clinical characteristics (Figures 6(a)–6(c)). We can draw the same conclusion for the first- and fifth-year samples (Figure S3). Similarly, the prognostic model of DFS was used to compare with stage, gender, age, and TNM in the first, third, and fifth years. The AUCs of the prognostic model of DFS in the training set, the test set, and the entire dataset were overall higher than those of other clinical characteristics (Figures 6(d)–6(f) and Figure S4). These results showed that the prognostic model of OS and DFS possessed better prognostic ability than the clinical characteristics.

**Figure 7:**
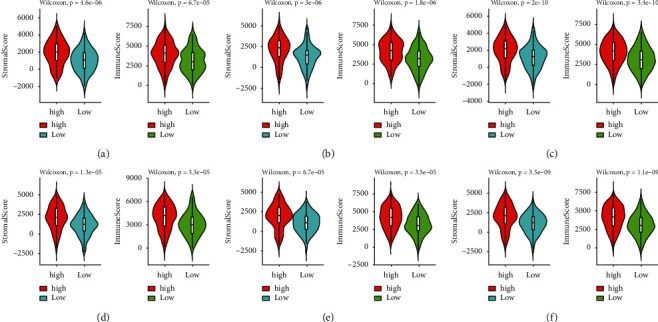
Difference of the stromal score and the immune score between the high- and low-risk group in OS in the training set (a), test set (b), and entire set (c). The difference of the stromal score and the immune score between the high- and low-risk groups in DFS (d–f).

Stromal cells are crucial components of TME, and the proportion of stromal cells in TME represents the stromal score. The tumor immune microenvironment plays a key role in the development of numerous cancers. The prognostic models of OS and DFS were built based on the RNA-seq data, for which the immune score and the stromal score for each sample can also be generated, using the estimate R package. For prognostic models of OS and DFS, immune scores and stromal scores of the high-risk group were significantly higher than those of the low-risk group in the training set, test set, and entire dataset (*P* value <0.05, Wilcoxon test) (Figure 7).

## 4. Discussion

lncRNAs have been found to play an important role in many biological processes, including the onset and development of cancer [28–32], which intuitively could serve as prognostic markers for cancers. In this study, we leveraged the TCGA RNA-seq data to build prognostic lncRNA models to evaluate the clinical outcomes of LUSC patients [33]. We first screened the differentially expressed lncRNAs, and then, we picked up those with a significant prognostic value. Finally, we constructed two prognostic models using LASSO-Cox regression analysis for OS and DFS, respectively. Ten lncRNAs were determined with significant contribution to the OS prognosis of LUSC, including *AC013457.1, AC124067.2, AP001189.1, AP002360.1, BANCR, LINC00519, LINC01807, MIR3945HG, FAM83A-AS1,* and *POU6F2-AS2*. For the DFS prognostic signature, 11 lncRNAs were identified, including *FAM83A-AS1, AC010275.1, AC015922.3, AL132712.1, LINC00261, LINC00511, LINC00519, LINC01980, TMEM99, MYOSLID*, and *NUP50-DT*.

Importantly, two lncRNAs, *FAM83A-AS1 and LINC00519*, were commonly identified as prognosticators for both OS and DFS analysis. *FAM83A-AS1* has been reported to be a key role in NSCLC. For instance, a study found that the overexpression of *FAM83A-AS1* increased *FAM38A* protein levels and induced the downstream *ERK1/2* phosphorylation in cells. Moreover, the overexpression of *FAM83A-AS1* promoted LUAD cell proliferation and invasion, which was consistent with our results [34]. Recent studies demonstrated that *LINC00519* was upregulated in LUSC, and silenced *LINC00519* prohibited proliferation, migration, invasion, and stimulated apoptosis in the LUSC cells [35]. Additionally, lncRNA *BANCR* in the OS model has been reported to function as an oncogene or tumor suppressor gene, which was often dysregulated in human cancers, including lung cancer [36, 37]. Meanwhile, lncRNA *MYOSLID* in the DFS model was closely related to important biological processes and pathways that regulate cancer metastasis [38–40]. Notably, the coefficients of the two lncRNAs are the second biggest among the panels.

We used prognostic and differentially expressed lncRNAs to construct predictive OS and DFS models that were superior to other clinical indicators. The method can be applied to other diseases or symptoms, once sufficient gene expression datasets are provided. Although these results had certain clinical significance, some limitations must be noted. The sample numbers of the patient and normal control are imbalanced in this study, which influence the machine learning results and lead to low AUC scores. A widely adopted method for imbalanced classification is resampling, which consists of picking up a subset of samples from the large group (undersampling) or bootstrapping samples from the small group (oversampling). For undersampling, the randomly chosen samples may be a biased sample set, resulting in an inaccurate representation of the population. Additionally, it can discard potentially useful information that could be important for model training. Unlike undersampling, oversampling leads to no information loss, but it increases the likelihood of overfitting because it replicates the samples in the small group.

Our models were built based on the public datasets and were not verified in external datasets. For clinical application and assessing the prognostic value of the proposed models, multicohort analysis integrating all the available LUSC expression data will be executed in our future studies. LINC00519 and FAM83A-AS1 were commonly identified as prognosticators in both models. The two lncRNAs have been reported to be associated with cell proliferation and invasion of lung cancer, suggesting the prognostic value of them, and further in vivo validation is warranted.

## Figures and Tables

**Table 1:** Clinical information of samples in the training set and test set.

Parameters	OS (*n* = 494)		DFS (*n* = 373)	
	Training (247)	Test (247)	Training (186)	Test (187)
*Age*				
>60	187	195	149	130
≤60	57	50	36	53

*Gender*				
Male	188	178	137	136
Female	59	69	49	51

*Stage*				
1 and 2	196	204	158	144
3 and 4	48	42	26	41

*PN*				
N0	161	156	124	109
N1–3	86	122	62	78

*PM*				
M0	206	200	154	150
M1–3	38	46	31	36

*PT*				
T0–2	196	205	158	143
T3-4	51	42	28	44

## Data Availability

The datasets used in this study are available in TCGA database.

## Supplementary Materials

Supplementary Materials Table S1. Correlation between the OS risk score and clinical characteristics. Table S2. Correlation between the DFS risk score and clinical characteristics. Figure S1. The optimal feature number of the LASSO-Cox regression model in the prognostic model of OS. Figure S2. The optimal feature number of the LASSO-Cox regression model in the prognostic model of DFS. Figure S3. Performance evaluation of the models and clinical characteristics. ROC curves of the OS prognostic model in year one, three, and five on different sets. Columns represent the observation year, while the rows represent the training set, test set, and entire set, respectively. Figure S4. Performance evaluation of the models and clinical characteristics. ROC curves of the DFS prognostic model in year one, three, and five on different sets. Columns represent the observation year, while the rows represent the training set, test set, and entire set, respectively.
