# Supplementary material for: Evaluation of the Prognostic Value of Long Noncoding RNAs in Lung Squamous Cell Carcinoma
Source: J Oncol. 2022 Jan 13;2022:9273628. doi: 10.1155/2022/9273628 (PMC8776467; doi:10.1155/2022/9273628)
Supplement: Supplementary Materials — Table S1. Correlation between the OS risk score and clinical characteristics. Table S2. Correlation between the DFS risk score and clinical characteristics. Figure S1. The optimal feature number of the LASSO-Cox regression model in the prognostic model of OS. Figure S2. The optimal feature number of the LASSO-Cox regression model in the prognostic model of DFS. Figure S3. Performance evaluation of the models and clinical characteristics. ROC curves of the OS prognostic model in year one, three, and five on different sets. Columns represent the observation year, while the rows represent the training set, test set, and entire set, respectively. Figure S4. Performance evaluation of the models and clinical characteristics. ROC curves of the DFS prognostic model in year one, three, and five on different sets. Columns represent the observation year, while the rows represent the training set, test set, and entire set, respectively. [file 9273628.f1.docx]

**Supplementary tables and figures**

**Table S1.** Correlation between OS risk score and clinical characteristics.

| Parameters | High-risk | Low-risk | PCC | P-value |
| --- | --- | --- | --- | --- |
| Training set | 123 | 124 |  |  |
| Age |  |  | 0.0329 | 0.6094 |
| >60 | 96 | 91 | 91 |  |
| <=60 | 25 | 32 | 32 |  |
| Gender |  |  | 0.1007 | 0.1144 |
| male | 87 | 101 | 101 |  |
| Famale | 36 | 23 | 23 |  |
| Stage |  |  | 0.1075 | 0.0939 |
| 1&2 | 95 | 101 |  |  |
| 3&4 | 26 | 21 |  |  |
| PN |  |  | 0.0966 | 0.13 |
| N0 | 78 | 83 |  |  |
| N1-3 | 45 | 41 |  |  |
| PM |  |  | 0.0081 | 0.9 |
| M0 | 101 | 105 |  |  |
| M1-3 | 20 | 18 |  |  |
| PT |  |  | 0.0919 | 0.1497 |
| T0-2 | 90 | 106 |  |  |
| T3-4 | 33 | 18 |  |  |
| Test set | 123 | 124 |  |  |
| Age |  |  | 0.0307 | 0.6324 |
| >60 | 101 | 94 |  |  |
| <=60 | 20 | 30 |  |  |
| Gender |  |  | -0.0315 | 0.6234 |
| male | 89 | 89 |  |  |
| Famale | 34 | 35 |  |  |
| Stage |  |  | -0.0942 | 0.1406 |
| 1&2 | 104 | 100 |  |  |
| 3&4 | 18 | 24 |  |  |
| PN |  |  | -0.0590 | 0.3557 |
| N0 | 78 | 47 |  |  |
| N1-3 | 45 | 77 |  |  |
| PM |  |  | 0.1444 | 0.0235 |
| M0 | 94 | 106 |  |  |
| M1-3 | 28 | 18 |  |  |
| PT |  |  | -0.0658 | 0.303 |
| T0-2 | 105 | 100 |  |  |
| T3-4 | 18 | 24 |  |  |
| Whole set | 248 | 246 |  |  |
| Age |  |  | 0.0324 | 0.4751 |
| >60 | 199 | 183 |  |  |
| <=60 | 45 | 62 |  |  |
| Gender |  |  | 0.0335 | 0.4573 |
| male | 177 | 189 |  |  |
| Famale | 71 | 57 |  |  |
| Stage |  |  | 0.0069 | 0.8774 |
| 1&2 | 200 | 200 |  |  |
| 3&4 | 46 | 44 |  |  |
| PN |  |  | 0.0203 | 0.6527 |
| N0 | 157 | 159 |  |  |
| N1-3 | 91 | 87 |  |  |
| PM |  |  | 0.0798 | 0.0774 |
| M0 | 197 | 209 |  |  |
| M1-3 | 48 | 36 |  |  |
| PT |  |  | 0.0122 | 0.7864 |
| T0-2 | 196 | 205 |  |  |
| T3-4 | 52 | 41 |  |  |

PCC, Pearson Correlation Coefficient.

**Table S2.** Correlation between the DFS risk score and clinical characteristics.

| Parameters | High risk | Low risk | PCC | P value |
| --- | --- | --- | --- | --- |
| Training set | 93 | 93 |  |  |
| Age |  |  | 0.1043 | 0.1576 |
| >60 | 75 | 74 |  |  |
| <=60 | 17 | 19 |  |  |
| Gender |  |  | 0.0233 | 0.7522 |
| male | 67 | 70 |  |  |
| Famale | 26 | 23 |  |  |
| Stage |  |  | -0.0546 | 0.4616 |
| 1&2 | 82 | 76 |  |  |
| 3&4 | 10 | 16 |  |  |
| PN |  |  | 0.0204 | 0.7826 |
| N0 | 64 | 60 |  |  |
| N1-3 | 29 | 33 |  |  |
| PM |  |  | 0.0667 | 0.3667 |
| M0 | 77 | 77 |  |  |
| M1-3 | 15 | 26 |  |  |
| PT |  |  | 0.0614 | 0.4050 |
| T0-2 | 78 | 80 |  |  |
| T3-4 | 15 | 13 |  |  |
| Test set | 93 | 94 |  |  |
| Age |  |  | 0.0584 | 0.4322 |
| >60 | 69 | 61 |  |  |
| <=60 | 22 | 31 |  |  |
| Gender |  |  | 0.0831 | 0.2583 |
| male | 65 | 71 |  |  |
| Famale | 28 | 23 |  |  |
| Stage |  |  | -0.0152 | 0.8377 |
| 1&2 | 72 | 72 |  |  |
| 3&4 | 20 | 21 |  |  |
| PN |  |  | -0.0664 | 0.3668 |
| N0 | 59 | 50 |  |  |
| N1-3 | 34 | 44 |  |  |
| PM |  |  | 0.0550 | 0.4561 |
| M0 | 74 | 76 |  |  |
| M1-3 | 18 | 18 |  |  |
| PT |  |  | 0.0019 | 0.9791 |
| T0-2 | 68 | 75 |  |  |
| T3-4 | 25 | 19 |  |  |
| Whole set | 186 | 187 |  |  |
| Age |  |  | 0.0768 | 0.1413 |
| >60 | 142 | 137 |  |  |
| <=60 | 40 | 49 |  |  |
| Gender |  |  | 0.0539 | 0.2993 |
| male | 133 | 140 |  |  |
| Famale | 53 | 47 |  |  |
| Stage |  |  | -0.0256 | 0.6236 |
| 1&2 | 153 | 149 |  |  |
| 3&4 | 31 | 36 |  |  |
| PN |  |  | -0.0175 | 0.7358 |
| N0 | 123 | 110 |  |  |
| N1-3 | 63 | 77 |  |  |
| PM |  |  | 0.0632 | 0.2246 |
| M0 | 147 | 157 |  |  |
| M1-3 | 37 | 30 |  |  |
| PT |  |  | 0.0389 | 0.4535 |
| T0-2 | 144 | 157 |  |  |
| T3-4 | 42 | 30 |  |  |

PCC, Pearson Correlation Coefficient.


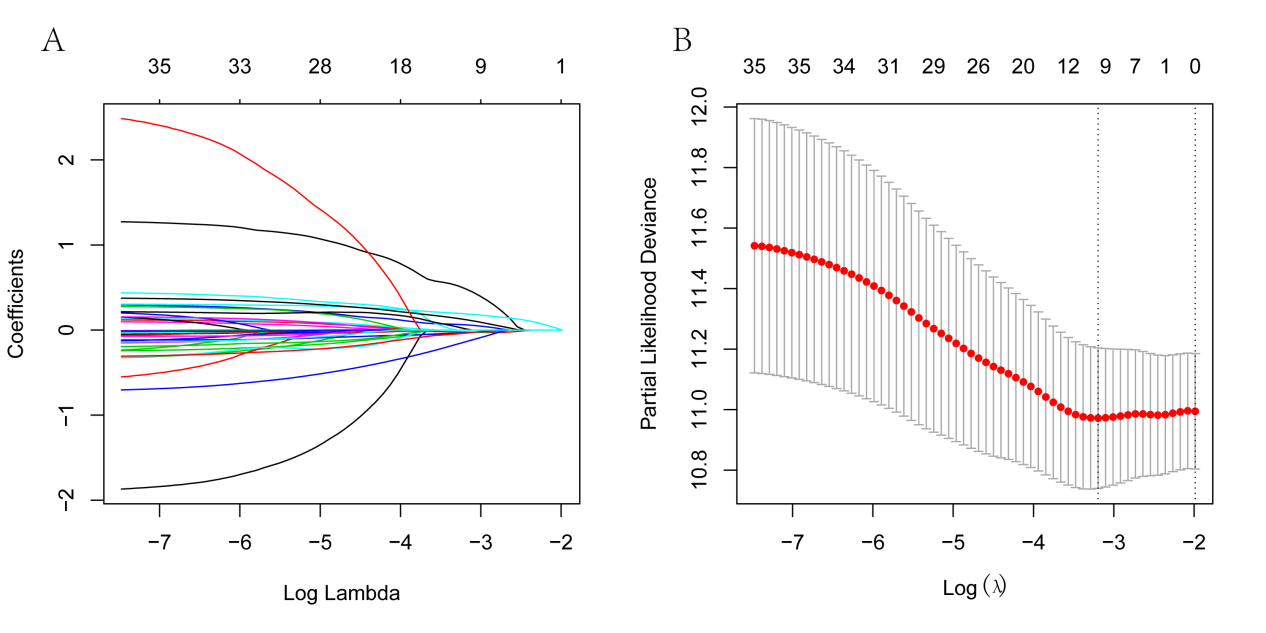


**Figure S1**. The optimal feature number of lasso Cox regression model in prognostic model of OS.


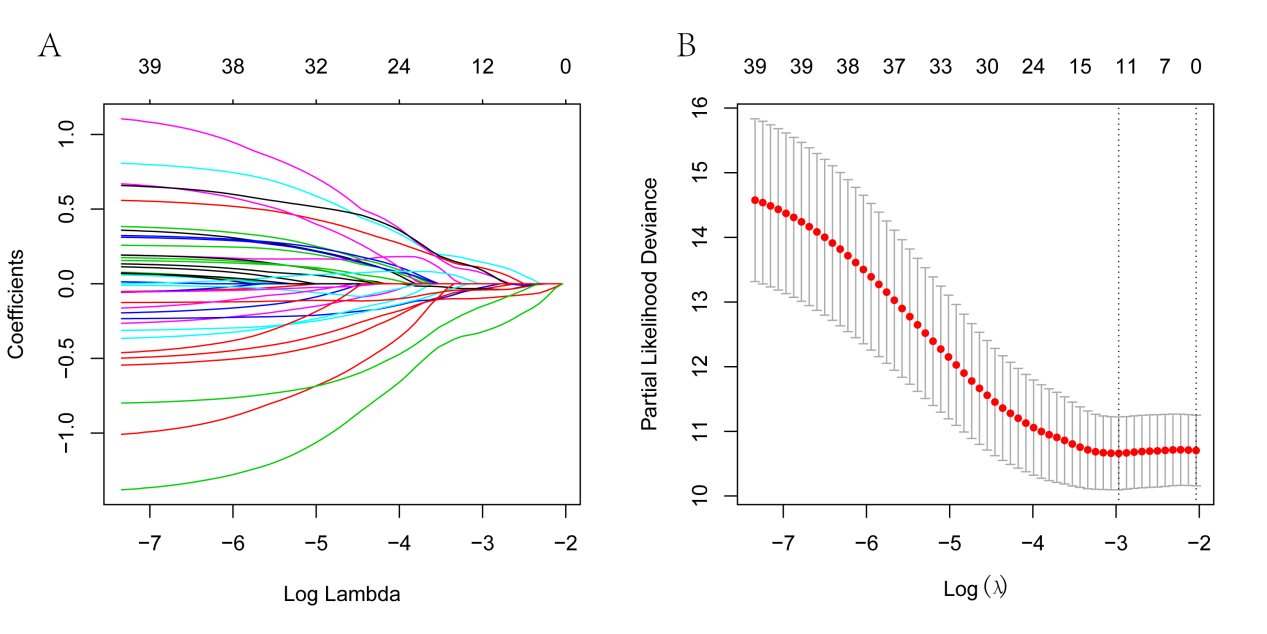


**Figure S2**. The optimal feature number of lasso Cox regression model in prognostic model of DFS.


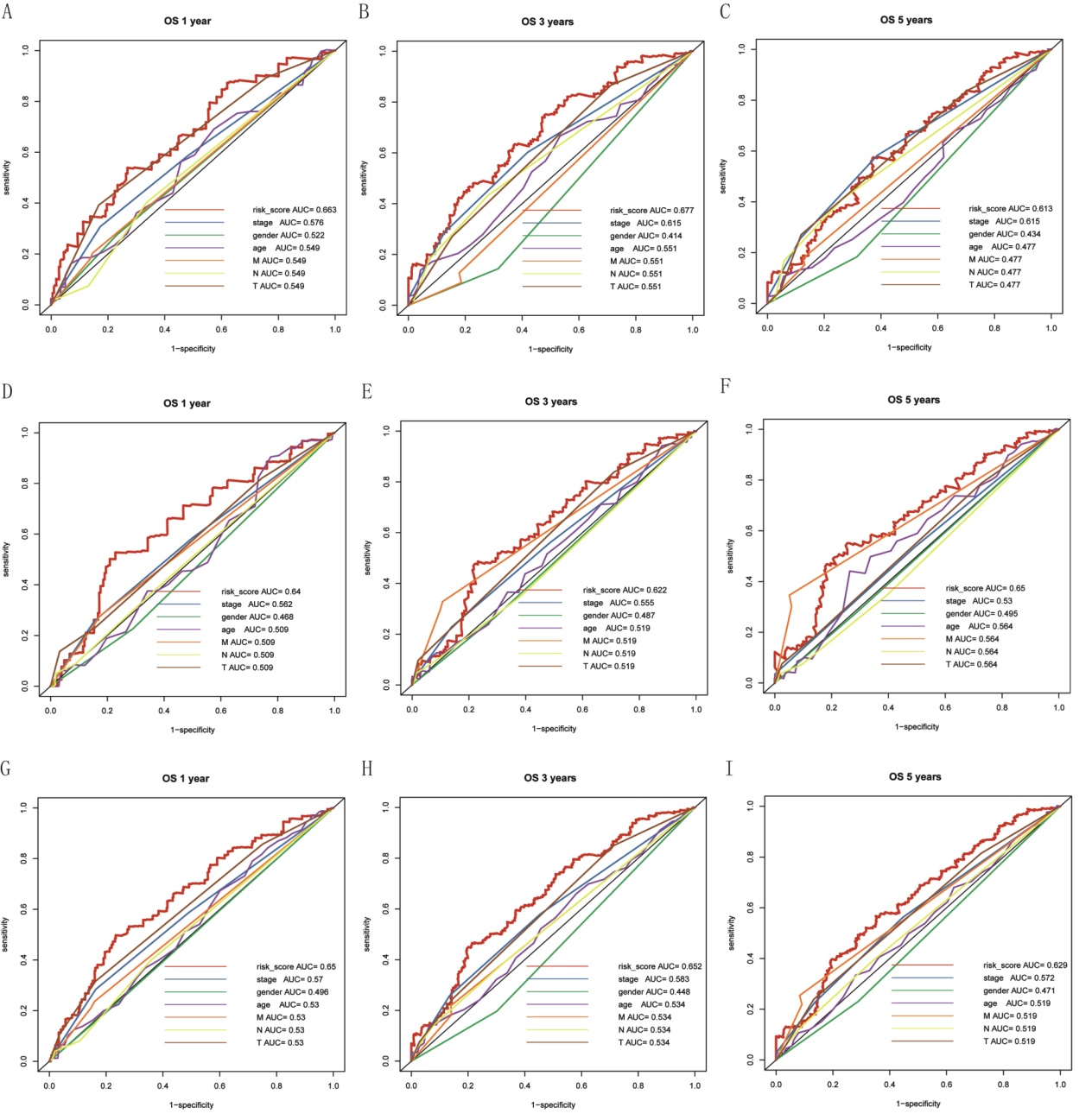


**Figure S3**. Performance evaluation of the models and clinical characteristics. ROC of OS prognostic model in year one, three, and five on different sets. Columns represent the observation year while the rows represent the training set, test set, and entire set, respectively.


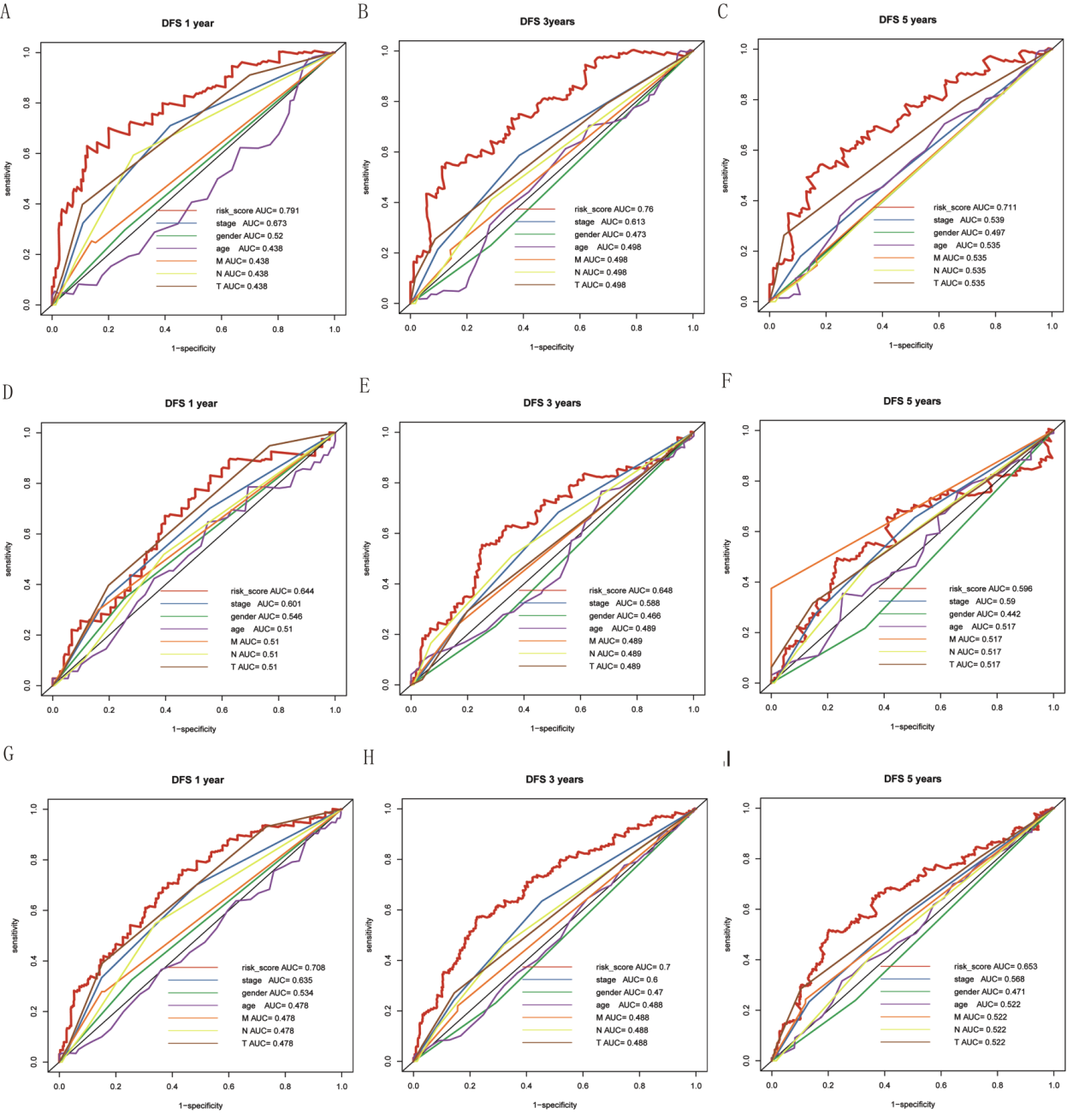


**Figure S4.** Performance evaluation of the models and clinical characteristics. ROC of DFS prognostic model in year one, three, and five on different sets. Columns represent the observation year while the rows represent the training set, test set, and entire set, respectively.
